# Glioma angiogenesis is boosted by ELK3 activating the HIF-1$\alpha$/VEGF-A signaling axis

**DOI:** 10.1186/s12885-023-11069-w

**Published:** 2023-07-14

**Authors:** Mou Yueyang, Hu Yaqin, Xue Guolian, Zhao Wenjian, Jiao Yang, Li Chen, Cao Haiyan, Chao Min, Deng Jianping, Dai Penggao, Zhu Hongli, Wang Liang

**Affiliations:** 1 College of Life Sciences, Northwest University, Xi’an, China; 2 Departments of Neurosurgery, Tangdu Hospital, Air Force Medical University, Xi’an, China

**Keywords:** Glioma, Angiogenesis, ELK3, VEGF-A, HIF-1$\alpha$

## Abstract

**Background:**

Clinical studies have shown that first-line use of anti-angiogenetic therapy can prolong progression-free survival but little progress has been made in extending the overall survival of the patients. We explored the role of ELK3 in glioma angiogenesis to improve and design more efficacious therapies.

**Methods:**

A tissue microarray and immunohistochemistry analysis were used to determine the expression of ELK3 protein in 400 glioma patients. Cell proliferation, metastasis, cell cycle, and apoptosis were monitored in U87 and U251 cells using CCK-8, EdU, transwell assays, and flow cytometry. A tube-formation assay, a rat aorta ring sprouting assay, and a matrigel plug assay were performed to examine the antiangiogenic activity of ELK3. An ELISA, Western blot, and correlation analysis of the CGGA dataset were used to detect the association between ELK3 and VEGF-A or ELK3 and HIF-1$\alpha$. Besides, orthotopic transplantation in nude mice and histopathological and immunological analysis of in vitro tumors were used to explore the effect of ELK3 on tumor progression and median survival.

**Results:**

ELK3 was upregulated in glioma tissues and associated with a poor prognosis. In vitro, ELK3 promoted cell proliferation and cell cycle progression, induced metastasis, and suppressed apoptosis. Then, silencing ELK3 inhibited VEGF-A expression and secretion by facilitating HIF-1$\alpha$ degradation via ubiquitination. Finally, knockdown ELK3 inhibited tumor progression and angiogenesis in vitro and in vivo, as well as prolonged nude mice’s median survival.

**Conclusions:**

Our findings first evidenced that ELK3 is crucial for glioma because it promotes angiogenesis by activating the HIF-1$\alpha$/VEGF-A signaling axis. Therefore, we suggest that ELK3 is a prognostic marker with a great potential for glioma angiogenesis and ELK3-targeted therapeutic strategies might hold promise in improving the efficacy of anti-angiogenic therapies.

## Introduction

In the central nervous system, $\sim 80\%$ of malignant brain tumors are gliomas, which are the most common primary intracranial tumors [1]. Notwithstanding surgery, radiotherapy, and chemotherapy all being used as part of the combined therapeutic approach, glioma still claims poor prognosis and is infamous for its high invasiveness, short survival time, and high 5-year mortality rates due to its high incidence, recurrence, and the formidable risk of recurrence [2]. Therefore, it cannot be more necessary to search for new targets and therapies for the treatment of gliomas.

Angiogenesis plays a key role in glioma and significantly correlates with tumor growth and progression [3]. Clinical studies have shown that first-line use of anti-angiogenetic therapy can prolong progression-free survival but little progress has been made in extending the overall survival of the patients [4]. VEGF-A (vascular endothelial growth factor-A), as a key molecular of angiogenesis, regulates micro-vessel density, and maintains the window of the choroid plexus endothelial cells [5, 6]. VEGF elevation may account for non-effective VEGF targeted therapies of gliomas, whose cause, however, is yet to be identified [7]. Hence, further and in-depth probes into the mechanism underlying its upstream regulation may help elucidate the mechanism of gliomas angiogenesis. As one of the many molecules that control VEGF-A, HIF-1$\alpha$ plays a pivotal role in tumor angiogenesis, embryonic angiogenesis, and the pathophysiology of ischemic disease [8, 9].

ELK3, also known as NET, is a transcriptional factor of the ETS (E-twenty-six) family and functions as a transcriptional repressor [10, 11]. Prior studies have demonstrated that the angiogenesis and cartilage ontology are correlated with ELK3 expression during mouse development, and that maintaining retinal artery integrity is one of the functions of ELK3 [11–13]. ELK3 expression in lymphatic endothelial cells is reported to be able to promote cancer progression and metastasis [14]. In addition, knockdown of ELK3 can severely impair tumor growth [15, 16]. All this suggests that ELK3 is directly involved in promoting tumor progression. However, there has been scant research on the role and function of ELK3 in glioma angiogenesis. In this sense, to develop and research novel and effective anti-angiogenetic therapeutic strategies for glioma, it cannot be more meaningful to uncover the role of ELK3 in angiogenesis.

It is against such a background that, using tissue microarray, we have found that ELK3 was upregulated in glioma and such upregulation was strongly related to poor prognosis of glioma. Moreover, this study indicates that ELK3 promotes the malignant progression of gliomas by promoting VEGF-A expression and inducing angiogenesis via facilitating HIF-1$\alpha$ ubiquitination. This study has revealed a novel role of the ELK3/HIF-1$\alpha$/VEGF-A axis in glioma progression and its underlying mechanism. In summary, ELK3 might be a potential prognostic marker for glioma angiogenesis and strategies targeting ELK3 may improve the efficacy of anti-angiogenesis therapies.

## Methods

### Clinical specimens and cell lines

Glioma tissues and para-carcinoma tissues matching the study were collected in surgical operations. All subjects gave their informed consent for inclusion before they participated in the study. The study was conducted in accordance with the Declaration of Helsinki, and the protocol was approved by the Institutional Review Board of Tangdu Hospital, Air Force Medical University. Human glioma cell lines, and normal human glial cell line were obtained from the Cell Bank of the Chinese Academy of Science (Shanghai, China). The stable ELK3-knockdown U87 cells were established according to the lentiviral vectors from Shanghai Jima Biotechnology’s instructions.

### Reverse transcription and qRT-PCR

TRIzol Reagent (Invitrogen, 15596018, Carlsbad, CA, USA) was used to extract total RNA from tissues and cells for real-time quantitative PCR. CDNA was synthesized as instructed on the reagent package (Prime ScriptTM RT Master Mix, Takara, RR036A, Shiga, Japan). SYBR Green-based qRT-PCR was used to examine mRNA expression levels. Primer sequences for RT-PCR are shown in the supplementary material.

### Western blot analysis

The collected cells and tissues were lysed using radioimmunoprecipitation assay (RIPA) lysis buffer (Beyotime, Nantong, China). Equal number of masses of proteins were separated by SDS-PAGE. Ultra-High Sensitivity ECL Kit (GK10008, GlpBio, CA, USA) was used to visualize the results. The density of the protein bands was semi-quantitatively analyzed using Image J software.

### Cell transfections

The cells were transfected at a density of $\sim 80\%$. The ELK3 overexpression plasmid and specific siRNA against ELK3 were synthesized by Genepharma (Shanghai, China). The transfection was performed with the transfection reagent Lipofectamine™ 2000 (Lip2000, Invitrogen, Carlsbad, CA, USA). The U87 cells were transfected with either ELK3-specific shRNA or control shRNA (Shanghai, China) for tumor formation in nude mice.

### Cell Counting Kit-8 assay

The cells were plated at 1$\times 10^3$ cells/well on 96-well plates with three wells for each group. Cell viability was assessed for over 6 days using Cell Counting Kit-8 (CCK-8) (Beyotime, Nantong, China). The absorbance was determined at 450 *nm*.

### EdU staining

For EdU staining, the treated cells were plated on laser confocal observation dishes and proliferated for 16-24 h. Meilun EdU Cell Proliferation Kit with Alexa Fluor 488 (Meilun Biotechnology, Ltd., Dalian, China) was used according to the manufacturer’s instruction.

### Flow cytometric analysis of apoptosis and the cell cycle

For apoptosis, the cells (1 $\times 10^6$cells) were suspended with 500 $\mu L$ binding buffer and with 5 $\mu L$ of Annexin V-FITC and 10 $\mu L$ of PI (FITC Annexin V Apoptosis Detection Kit I, Sigma Aldrich, St. Louis, USA) added, and were later mixed. Next, the suspended cells were incubated at 4$^{\circ }$C for 30 min in dark. For the cell cycle, the cells were suspended with 500 $\mu L$ of binding buffer and assayed by PI/RNase Staining Buffer (BD Biosciences, Franklin Lakes, NJ, USA) according to the manufacturer’s instruction. The apoptotic cells were analyzed using EXPO32 ADC, an analysis software (Beckman Coulter, Brea, CA, USA). The cell cycle was analyzed using ModFit 3.0 analysis software (Verity Software House, Inc. Topsham, ME, USA).

### Transwell assay

After 24 h of transfection, the cells (1$\times 10^5$ cells/well) were collected and resuspended in 100 *ml* of serum-free DMEM (PM150310, Procell, Wuhan, China) and seeded into the top chambers of 24-well transwell plates (Costar, CA, USA) coated with or without 100 $\mu L$ of Matrigel (BD Biosciences, Franklin Lakes, NJ, USA) and 600 $\mu L$ complete media containing added to the lower chamber. The cells were allowed to migrate for about 30 h (48 h coated with Matrigel). Observed under a Leica microscope (Leica, DM2500, Wetzlar, Germany) at a magnification of $\times$20.

### Immunohistochemistry (IHC) and immunofluorescence (IF)

Immunofluorescence (IF) was performed as described previously [16]. IHC staining of 4$\times$100 human glioma samples in tissue microarray (TMAs) and paraffin slides which were taken from nude mice tumor tissues were performed using anti-ELK3 antibodies. IHC staining of other proteins used corresponding antibodies. The TMAs immunostaining was analyzed by three independent researchers blinded to the clinical data.

### HUVEC tube formation assay

Tube formation assay was performed using a $\mu$-Slide Angiogenesis System ($\mu$-Slide Angiogenesis ibiTreat; 81506, Munich, Germany) coated with Matrigel. HUVECs were suspended in 50 $\mu L$ of supernatant retrieved from the glioma cells treated with siELK3. After 6 h of incubation, the cells were imaged at $\times$4 magnification under an inverted microscope (Leica DMI 6000B, Wetzlar, Germany).

### Matrigel plug assay

Matrigel plug assay was performed to evaluate properties of the angiogenesis and endothelial activation of ELK3 as described by prior studies [17].

### Animal experiments

The Center for Animal Experiments of Wuhan University (Wuhan, China) provided nude immunocompromised mice, and breeding was carried out under specific pathogen-free conditions. As prior studies described [18], there are shELK3 group and LV3 group for the U87 cell lines. The isolated U87 cells of every group were planted into the right lateral brain ventricle (AP = -2 mm, ML = -2 mm, DV = -3 mm, the anterior fontanelle as the origin). This study was approved by the Ethics Committee of the Second Affiliated Hospital of Air Force Medical University.

### Statistical analysis

Graphpad 9.0 was used to analyze the independent samples using 2-sided independent Student’s t-tests and to analyze the relationships across ELK3, VEGF-A, HIF-1$\alpha$ and the expression levels of those factors at the protein level and mRNA level. Cell counts, migration distance measurement, and Western blot relative quantitation were performed using Image J. All statistical results from the quantitative analysis of in vitro experiments are presented as means ± SEM, and *p* values < 0.05 were considered statistically significant.

## Results

### ELK3 is upregulated in glioma and correlated with poor prognosis

**Fig. 1:**
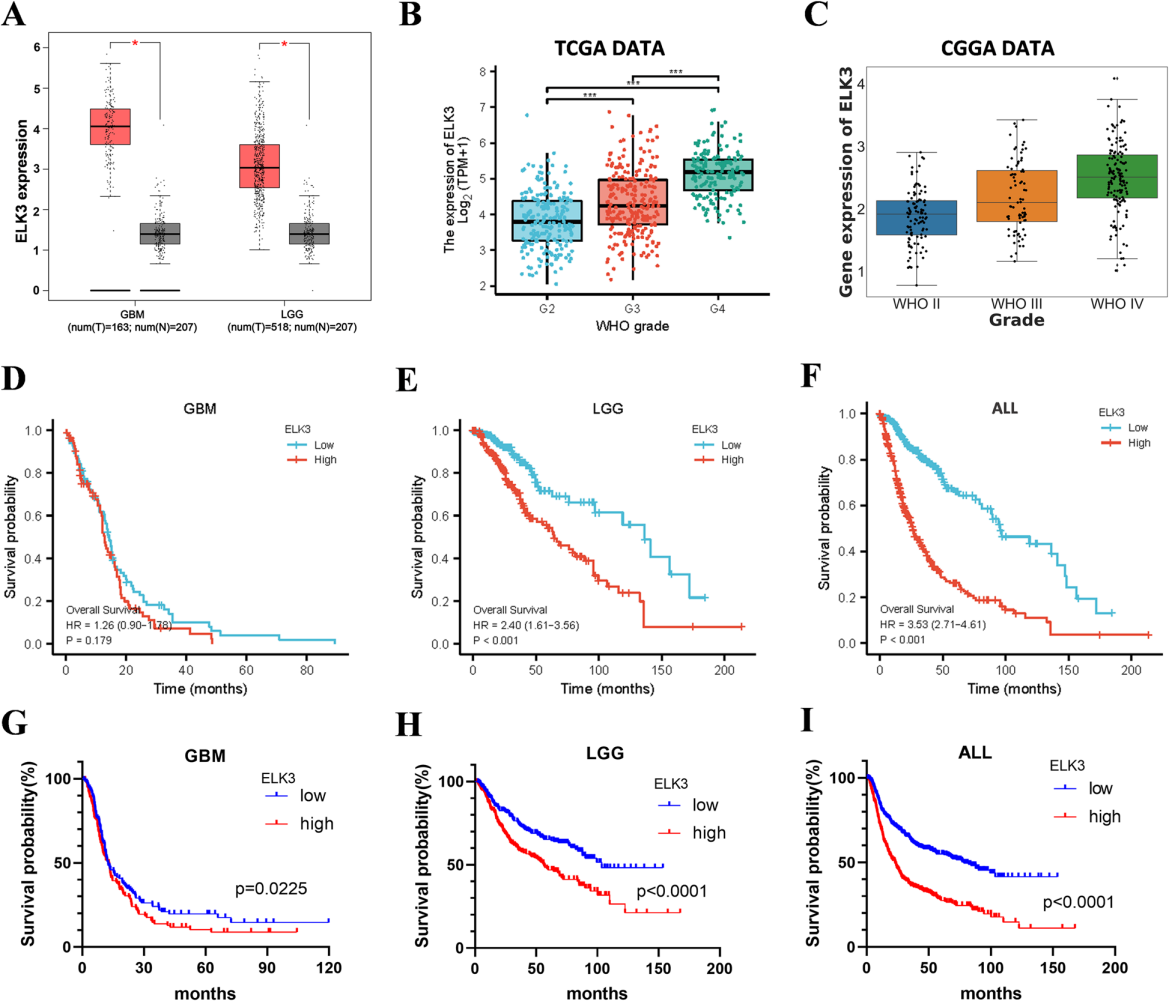
ELK3 is upregulated in glioma and correlated with poor prognosis.**A** Box plot of ELK3 gene expression levels in GBM or LGG (red box) and normal brain (gray box) in the GEPIA database (* p<0.05). **B** The expression of ELK3 in various grades of gliomas in the TCGA database. **C** The expression of ELK3 in various grades of gliomas in the CGGA database. **D**-**F** Kaplan-Meier survival analysis of the expression levels of ELK3 in GBM, LGG and all patients based on the TCGA data. **G**-**I** Kaplan-Meier survival analysis of the expression levels of ELK3 in GBM, LGG and all patients based on the CGGA data. *P<0.05, **P<0.01, ***P<0.001

The expression of ELK3 in gliomas and normal brain tissue (or paracancerous tissue) was analyzed in the GEPIA database. In GBM, ELK3 is significantly overexpressed in glioma patient tissues (n=163) compared to normal brain tissue (or paracancerous tissues) (n=207,p<0.05). In LGG, ELK3 is significantly overexpressed in glioma patient tissues (n=518) compared to normal brain tissue (or paracancerous tissues) (n=207,p<0.05) (Fig. 1A). The analysis results of TCGA and CGGA databases showed that ELK3 expression was the lowest in WHO II gliomas, the higher in WHO III gliomas, and the highest in WHO IV gliomas (Fig. 1B-C). These results indicate that ELK3 is highly expressed in gliomas, and its expression is gradually upregulated as the malignancy of gliomas increases.

To investigate the relationship between ELK3 expression and survival in glioma patients, TCGA and CGGA databases were used to analyze the survival of patients under high and low ELK3 expression conditions in LGG and GBM. The TCGA database shows that in LGG, compared to patients with low ELK3 expression, patients with high ELK3 expression had a significantly reduced median survival time (66 months vs. 138 months, p<0.001) (Fig. 1E). Although there was no significant difference in median survival time between patients with high and low ELK3 expression in GBM patients in the TCGA database (Fig. 1D), among all gliomas, patients with high ELK3 expression had significantly shorter median survival time compared to those with low ELK3 expression (28 months vs. 95 months, p<0.001). Meanwhile, CGGA data analysis showed that in GBM, the survival time of patients in the high ELK3 group was significantly reduced compared to those in the low ELK3 group (11.27 months vs 12.6 months, p=0.0225) (Fig. 1G). In LGG, the survival time of patients in the high ELK3 group was significantly reduced compared to the low ELK3 group (56 months vs.103.6 months, p<0.0001) (Fig. 1H). In all grades of gliomas, the median survival time was 22.8 months in the high ELK3 group and 78.4 months in the low ELK3 group (p<0.0001) (Fig. 1I). These results indicate that high expression of ELK3 predicts poor prognosis in patients.

**Fig. 2:**
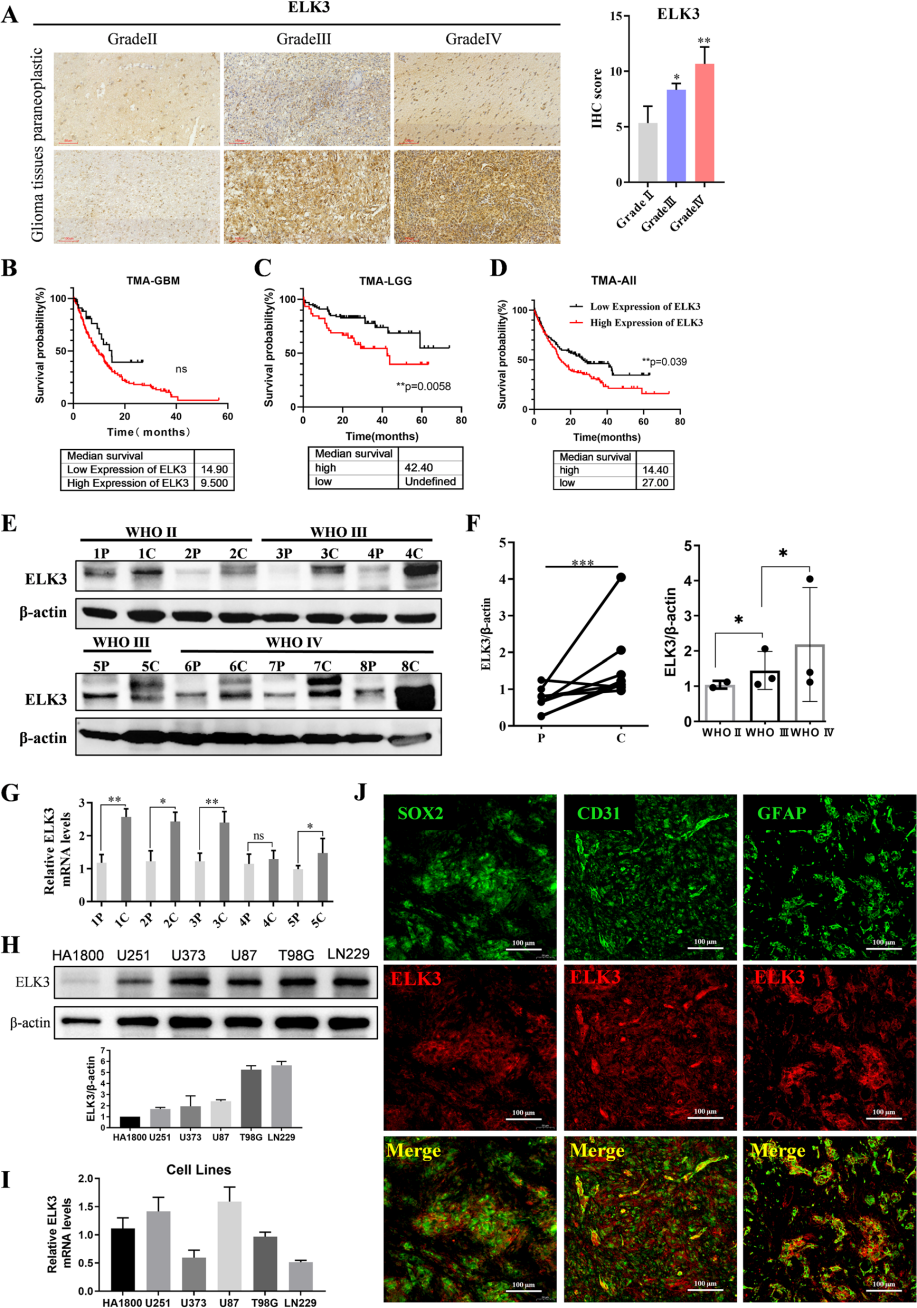
ELK3 is upregulated in glioma and correlated with poor prognosis. **A** Representative immunohistochemical staining of ELK3 in Tissue Microarray (TMA) data, scale bar: 100$\mu m$. **B**-**D** Kaplan-Meier survival analysis of the expression levels of ELK3 in GBM, LGG and all patients based on the microarray data. **E** ELK3 expression was determined in randomly selected 2 pairs of WHO II, 3 pairs of WHO III, and 3 pairs of WHO IV of glioma tissues and compared paraneoplastic tissues by Western blot. **F** Statistical results of ELK3 expression in different grades of gliomas and adjacent tissues. **G** The expression of ELK3 was determined in 5 pairs of glioma tissues and compared paraneoplastic tissues by qRT-PCR. **H**-**I** Evaluate the Western blot and qRT PCR results of ELK3 expression in glioma cell lines (U251, U373, U87, T98G, LN229) and HA1800 cell lines. **J** ELK3(red) and SOX2, CD31, GFAP (green) double immunofluorescence. All *p* values resulted from 2-sided statistical test, ns: not significant, *P<0.05, **P<0.01, ***P<0.001, ****P<0.0001

To further verify whether ELK3 is involved in gliomas, using the tissue microarrays (TMAs) to investigate ELK3 expression level in gliomas. As shown in Fig. 2A, ELK3 expression increased significantly in tumor tissues compared to paraneoplastic tissues, and was progressively upregulated with the increase in malignancy of glioma. To further verify whether increased ELK3 expression is associated with poor prognosis in glioma patients, all patients were divided into LGG group, GBM group, and ALL patient group based on the degree of malignancy of the tumor. Immunohistochemical scores>6 was used as high ELK3 group, and immunohistochemical scores $\le$ 6 were used as low ELK3 group. Kaplan Meier curves were used to analyze the survival curves of high ELK3 and low ELK3 in LGG, GBM, and All gliomas, respectively. The results showed that in LGG patients, compared with the ELK3 low expression group (n=48, median survival time greater than 80 months), the ELK3 high expression group (n=101, 42 months) had significantly shorter median survival time for glioma patients (p=0.0058); In GBM patients, compared with the ELK3 low expression group (n=100, 14.9 months), the ELK3 high expression group (n=37, 9.5 months) had a shorter median survival time for glioma patients; In the all TMA glioma samples, compared with the ELK3 low expression group (n=143, 27 months), the ELK3 high expression group (n=143, 14.4 months) significantly reduced the median survival of glioma patients (p=0.039). The comparison showed that a high level of ELK3 expression in gliomas has been associated with a poor prognosis (Fig. 2B-D). Next, ELK3 protein levels were also confirmed by Western blot assays on randomly selected 2 pairs of WHO II, 3 pairs of WHO III, and 3 pairs of WHO IV tumor tissues and their corresponding non-tumor tissues, which further confirmed the increase in ELK3 protein expression in glioma tissues (Fig. 2E-F). A qRT-PCR was also used to confirm an increase in ELK3 mRNA levels in glioma tissues compared to non-tumor tissues in five randomly matched tumor samples (Fig. 2G). Besides, in glioma cell linesand the normal human glial cell line HA1800 cells, ELK3 expression was determined by Western blot. ELK3 protein expression was found to have increased in glioma cells lines (Fig. 2H). An increase in ELK3 mRNA was also detected in U251 and U87 cell lines (Fig. 2I). Additionally, immunofluorescence assay revealed that ELK3 had co-localized with GFAP, SOX2 or CD31, indicating that it was highly expressed in stem cells, astrocytes and blood vessels of glioma tissues from glioma patients (Fig. 2J). These results imply that ELK3 is upregulated in glioma and correlated with poor prognosis, indicating that it plays a crucial role in glioma progression.

### ELK3 has a vital effect on malignant phenotype in vitro

**Fig. 3:**
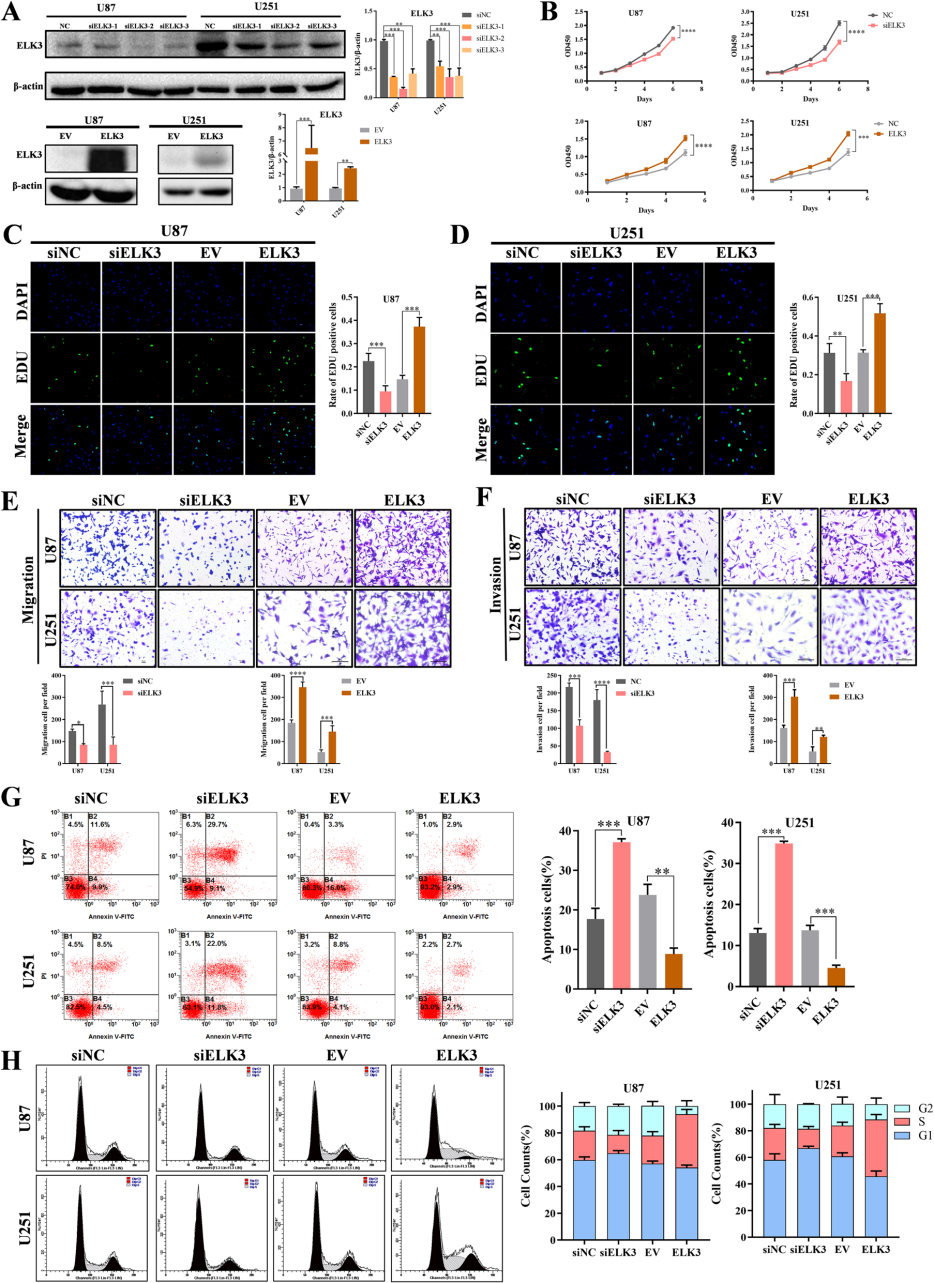
Effect of ELK3 on malignant phenotype in glioma cell lines. **A** Western blot analysis of ELK3 expression after transfection with siELK3 oligos and ELK3 overexpression plasmid in U87 and U251 cells. **B**-**D** Cell proliferation was detected by CCK-8 assays and EDU assays in U87 and U251 cells with ELK3 knockdown or overexpression, respectively (n = 3, **P<0.01, ***P<0.001, ****P<0.0001, two-way ANOVA with post hoc test). **E**,**F** Effects of ELK3 in U87 and U251 cells on metastasis in vitro using transwell migration assay and transwell invasion assay, scale bar: 100$\mu m$. **G** Apoptosis was analyzed by flow cytometry Annexin V/PI assay in U87 and U251 cells transfected with control siRNA, siELK3, or EV (empty vector) and ELK3 overexpression plasmid. **H** Cell cycle was analyzed by flow cytometric in U87 and U251 cells transfected with control siRNA, siELK3, or EV and ELK3 overexpression plasmid. All experiments were repeated at least three times, 2-sided statistical test, ns: no significant (*P<0.05, **P<0.01, ***P<0.001, ****P<0.0001)

Based on the expression profile and characterization of ELK3 in glioma cell lines, the effect of ELK3 on the phenotype of glioma cells in U87 and U251 cell lines was detected by silencing or overexpressing ELK3 (Fig. 3A). CCK-8 assay results demonstrated that cell proliferation was markedly inhibited after ELK3 was silenced, but was promoted after ELK3 was overexpressed (Fig. 3B). Also, the EdU assay showed that overexpression of ELK3 stimulated cell proliferation when ELK3 was silenced (Fig. 3C-D). Next, the role of ELK3 in glioma metastasis was examined by evaluating cell migration and invasion. Likewise, the transwell assay showed that ELK3 knockdown significantly reduced migration and invasion of glioma cells, but increased cell migration and invasion after ELK3 is overexpressed (Fig. 3E-F). Furthermore, silencing of ELK3 increased cell apoptosis, while overexpression of ELK3 inhibited cell apoptosis in glioma cells (Fig. 3G). The potential role of ELK3 in cell cycle progression was also examined. The results of flow cytometry evidenced that silencing ELK3 inhibited cell cycle progression, and high ELK3 expression promoted cell cycle progression (Fig. 3H). To be brief, these results imply that ELK3 has a vital effect on malignant phenotype in glioma cell lines.

### Silencing ELK3 inhibits angiogenesis in vitro and in vivo

**Fig. 4:**
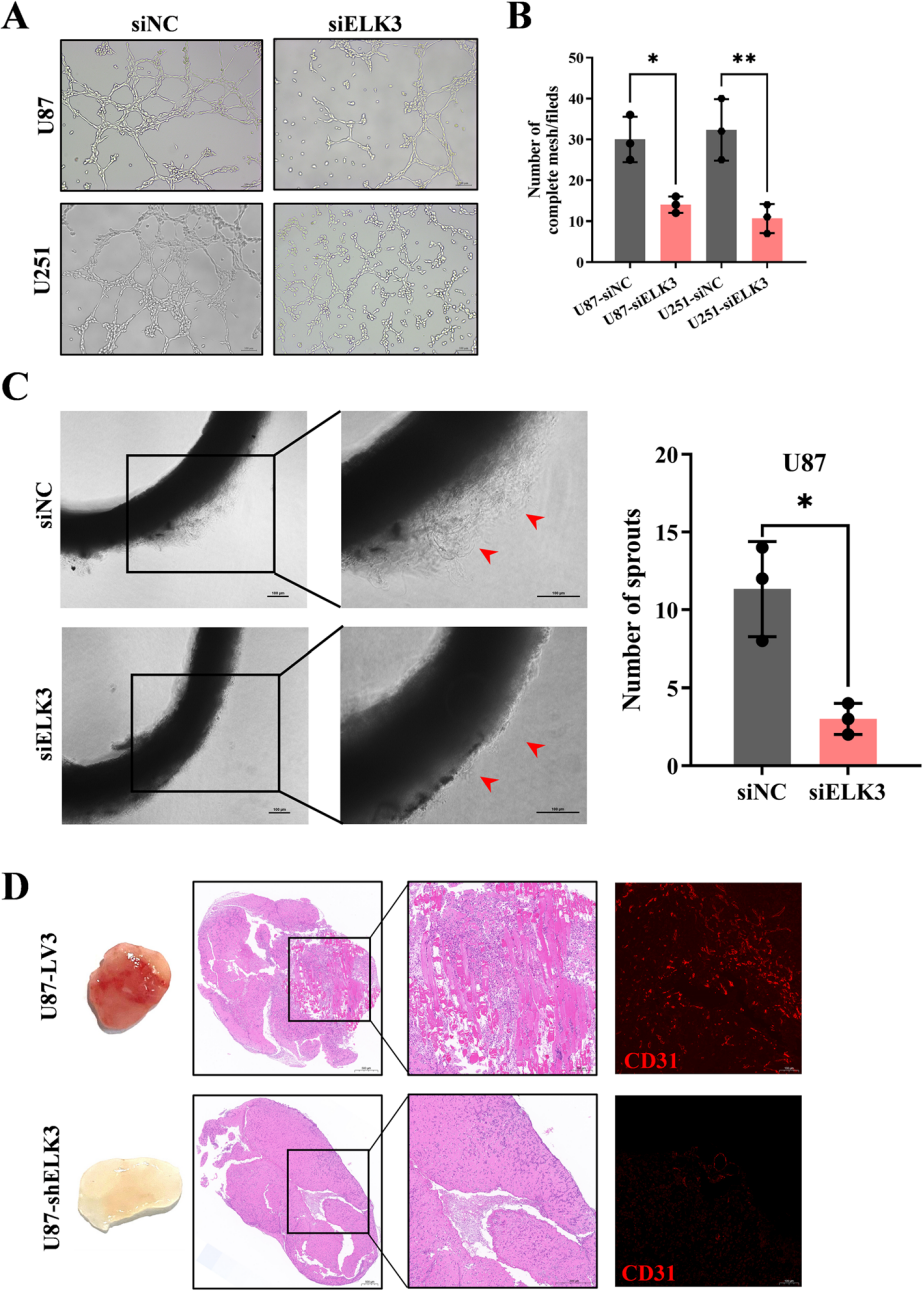
Silencing ELK3 inhibits angiogenesis in vitro and in vivo. **A** Tube formation in HUVEC cells was treated with supernatants from U87 and U251 cells transfected with control siRNA or siELK3. **B** The number of complete meshes per visual field was counted, scale bar: 100$\mu m$. **C** Representative Matrigel plugs are on the left, and corresponding HE and CD31 immunohistochemical staining were performed on the right. **D** Rat aortic ring assay shows that compared with siNC, supernatants from U87 transfected with siELK3 significantly suppressed the capillary sprouting ability of rat arterial rings, scale bar:100$\mu m$, right: number of sprouts. All experiments were repeated at least three times, Data are expressed as mean ± SD (*P<0.05, **P<0.01)

Prior studies found that ELK3 expression correlates with angiogenesis remarkably. Combined with the result shown in Fig. 2J, co-localization of ELK3 with CD31 in glioma tissues indicated that ELK3 may be highly expressed around blood vessels in glioma tissues. To further investigate the role of ELK3 in angiogenesis, the effect of ELK3 on endothelial network formation of HUVECs was further determined in vitro via tubule formation assay. U87 and U251 cells were transfected by siELK3 and incubated for 24 h. Then the transfection supernatant was used to culture HUVECs in ibidi $\mu$-Slides Angiogenesis System coated with a layer of gel matrix of 0.8 mm thick. Tube formation assays indicated that silencing of ELK3 suppressed tube formation of endothelial cells than those transfected by siNC (Fig. 4A-B). To observe the changes in angiogenic indexes in vivo, Matrigel plug assay was performed, and the dissected Matrigel plugs were stained with HE and CD31 immunofluorescence. Results of the plug assay showed that the plugs from the group treated with ELK3 knockdown cells had significantly fewer blood vessels than the LV3 group (Fig. 4C). Moreover, rat arterial ring assays revealed that ELK3 knockdown exhibited a weaker ability to promote the outgrowth of arterial rings than the LV3 empty vector, indicating that silencing ELK3 may inhibit vasculature generation (Fig. 4D). These results indicate that silencing ELK3 inhibits angiogenesis both in vitro and in vivo.

### VEGF-A is a downstream molecule crucial for ELK3

**Fig. 5:**
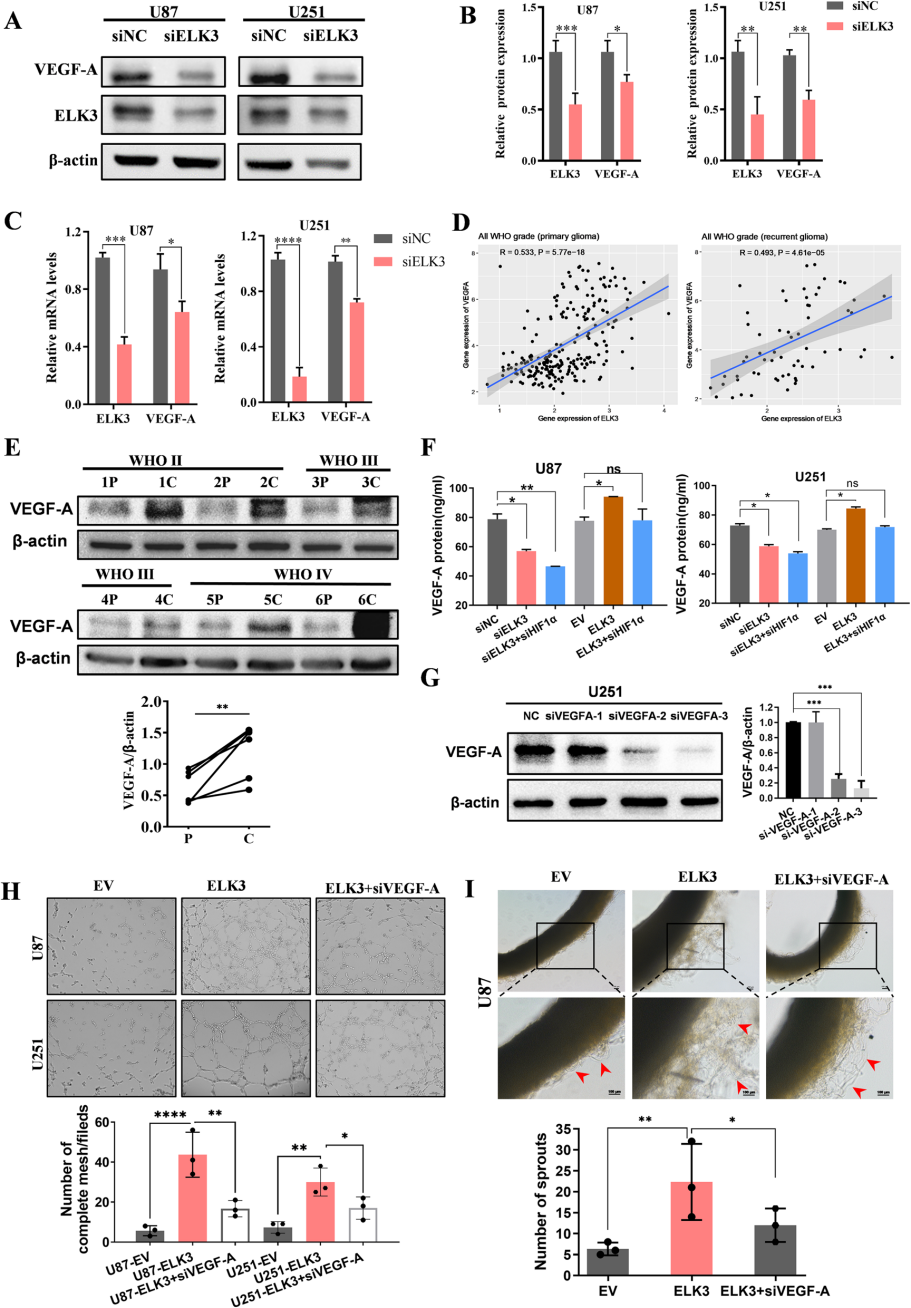
VEGF-A is a downstream molecule critical for ELK3. **A** Western blotting was used to confirm a significant reduction in VEGF-A expression after silencing ELK3 in U87 and U251 cells. **B** Quantified graph of A. **C** qRT-PCR was used to confirm a significant reduction mRNA expression of VEGF-A after silencing ELK3 in U87 and U251 cells. **D** Correlation between HIF-1$\alpha$ and ELK3 mRNA expression on CGGA database. **E** The expression of VEGF-A was determined in 6 pairs of glioma tissues and compared with paraneoplastic tissues by Western blot. **F** The VEGF-A concentration of the cellular supernatant was measured by VEGF-A ELISA kits. The cellular supernatant was taken from U87 and U251 cells transfected with control siRNA or siELK3. **G** Western blot results of knockout efficiency of three siVEGF-A types. **H** Tube formation in HUVEC cells was treated with supernatants from U87 and U251 cells transfected with ELK3 or ELK3+siVEGF-A. **I** Effects of overexpression of ELK3 and simultaneous knockdown of VEGF-A in U87 supernatant on capillary sprouting ability in rat arterial rings, bar:100$\mu m$. All experiments were repeated at least three times. Data are expressed as mean ± SD, ns: not significant, *P<0.05, **P<0.01, ***P<0.001, ****P<0.0001

To further investigate the mechanism underlying the ELK3 regulation of angiogenesis, Western blot assay was performed to verify whether ELK3 can effectively regulate VEGF-A. The Western blot analysis showed that knockdown of ELK3 significantly inhibited VEGF-A expression in glioma cells (Fig. 5A-B). Results of real-time quantitative PCR confirmed these results and demonstrated that knockdown of ELK3 inhibited the increase of VEGF-A at transcriptional level (Fig. 5C). Besides, in all WHO grade (primary glioma), the correlation coefficient between ELK3 and VEGF-A was R=0.533, P<0.001; In all WHO grade (recurrent glioma), the correlation coefficient between ELK3 and VEGF-A is R=0.493, P<0.001; Indicating a positive correlation between ELK3 and VEGF-A expression in gliomas, even if the correlation coefficient is not high enough (Fig. 5D). In addition, six pairs of tumor tissues and their corresponding non-tumor tissues were randomly selected for Western blot analysis, which further confirmed the increase in the expression of VEGF-A protein in glioma tissues. This expression pattern is consistent with the expression trend of ELK3 in glioma tissues (Fig. 5E). Generally, VEGF-A exerts its influence by secreting itself into the extracellular space and subsequently binds itself to its receptors on the endothelial cells to activate downstream signaling pathways. In addition, we measured VEGF-A production in supernatants from U87 and U251 cell lines by using ELISA, which showed that the concentration of VEGF-A in the culture medium of siELK3 treatment cells was lower than that in the negative control, whereas the concentration of VEGF-A in the overexpressed ELK3 cells was higher than the negative control (Fig. 5F). In addition, we tested the effect of overexpression of ELK3 on the ability of cell vascular analogue formation by HUVEC tube formation experiments. The results showed that overexpression of ELK3 promoted HUVEC tube formation stimulated by conditioned medium, and this promoting effect could be rescued by siVEGF-A treatment (Fig. 5G-H). At the same time, in vitro culture experiments on rat aortic rings have shown that overexpression of ELK3 can promote vascular sprouting in arteries, which can be rescued by knocking down VEGF-A (Fig. 5I). These results indicated that ELK3 had induced expression and production of VEGF-A in glioma cells. Thus, it can be inferred that VEGF-A is a downstream molecule crucial for ELK3.

### ELK3 regulates VEGF-A by interacting with HIF-1$\alpha$

**Fig. 6:**
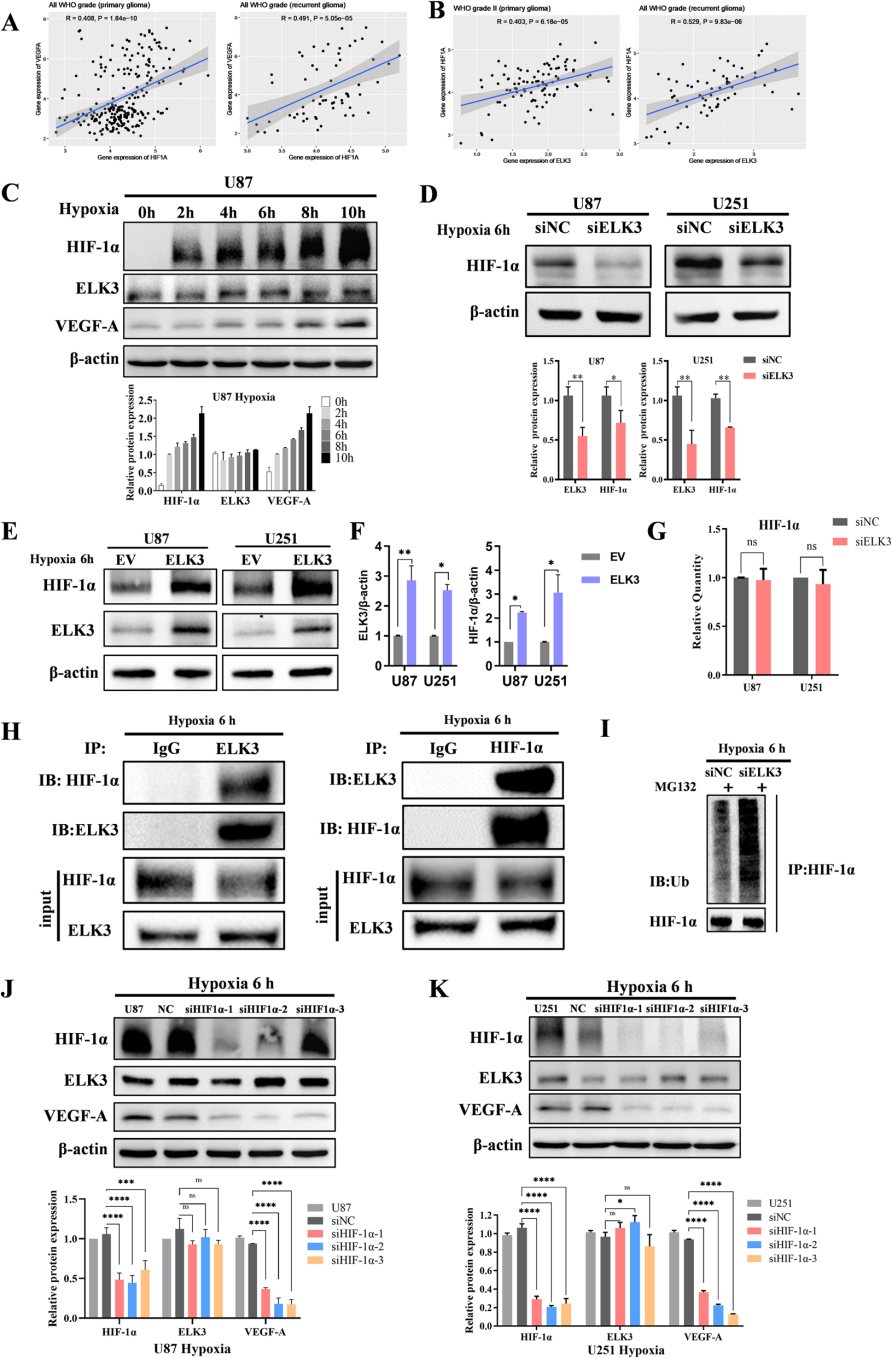
ELK3 regulates VEGF-A by interacting with HIF-1$\alpha$. **A**,**B** Correlation between HIF-1$\alpha$ and VEGF-A, HIF-1$\alpha$ and ELK3 mRNA expression on CGGA database. **C** The expressions of HIF-1$\alpha$, VEGF-A and ELK3 were detected by Western blotting under different hypoxia times. **D** The protein expression results of HIF-1$\alpha$ after silencing ELK3 in U87 and U251 cells. **E**,**F** The protein expression of HIF-1$\alpha$ after overexpressing ELK3 in U87 and U251 cells. **G** qRT-PCR was used to detect mRNA expression of HIF-1$\alpha$ after silencing ELK3 in U87 and U251 cells. **H** ELK3 and HIF-1$\alpha$ interaction is confirmed using a reciprocal Co-Immunoprecipitation (Co-IP) experiment. **I** Polyubiquitination levels of HIF-1$\alpha$ after knockdown ELK3. **J**,**K** WB assay showed that knockdown of HIF under hypoxia reduced the expression of VEGF-A, but ELK3 expression remained unchanged in U87 and U251 cells. All *p* values resulted from 2-sided statistical test, ns: not significant, and all experiments were repeated at least three times. (*P<0.05, **P<0.01, ***P<0.001, ****P<0.0001)

The above experiments revealed that knockdown of ELK3 inhibited VEGF-A at transcriptional level. HIF-1$\alpha$, a transcription factor that involved in VEGF-A expression, has been implicated in its regulation [19]. A study by LEE et al. [20] found that ELK3 was involved in migration and invasion by regulating HIF-1$\alpha$ signaling pathway in liver cancer stem cells. Based upon that finding, in this study, we speculated that ELK3 can regulate VEGF-A by interacting with HIF-1$\alpha$ in glioma cells. An analysis of CGGA revealed that in all WHO grades (primary glioma), there is a positive correlation between HIF-1$\alpha$ and VEGF-A (R=0.408, P<0.001); In recurrent glioma, there is also a positive correlation between HIF-1$\alpha$ and VEGF-A (R=0.491, P<0.001) (Fig. 6A). Similarly, there is a positive correlation between ELK3 and HIF-1$\alpha$ (correlation coefficients R=0.403, R=0.529, respectively, and P<0.001), indicating a positive correlation between the expression of ELK3 and HIF-1$\alpha$, even if the correlation coefficient is not high enough (Fig. 6B). It has been accepted that HIF-1$\alpha$ is abundantly expressed in cells treated with hypoxia (O$_{2}$, 1$\%$), whereas no HIF-1$\alpha$ was detected in normoxia cells [21]. To elucidate the relationship between ELK3 expression and HIF-1$\alpha$ expression, we first examined the level of HIF-1$\alpha$ expression under hypoxic condition at 2, 4, 6, 8 and 10 h. Results of Western blot assay indicated that HIF-1$\alpha$ and VEGF-A expression increased significantly with prolonged hypoxia duration whereas ELK3 expression remained unchanged with prolonged hypoxia duration (Fig. 6C). Combined with the above results and cell states at different hypoxia time points, hypoxia for 6 h was used as the treatment condition. In addition, the finding that silencing of ELK3 decreased HIF-1$\alpha$ expression, while overexpression of ELK3 promoted HIF-1 expression under hypoxic condition in glioma cells, suggests that ELK3 can regulate HIF-1$\alpha$ (Fig. 6D-F). PCR results indicated that HIF-1$\alpha$ was decreased only at protein level but not at mRNA level, suggesting a post-transcriptional regulation (Fig. 6G). An immunoprecipitation analysis approach demonstrated an interaction between ELK3 and HIF-1$\alpha$ protein (Fig. 6H). Knockdown of ELK3 substantially increased the level of HIF-1$\alpha$ ubiquitination (Fig. 6I). These results suggest that silencing ELK3 can transcriptionally inhibit VEGF-A expression and secretion by facilitating HIF-1$\alpha$ degradation via ubiquitination. Moreover, Western blot analysis indicated that knockdown of HIF-1$\alpha$ inhibited VEGF-A expression in hypoxia whereas ELK3 expression remained intact in the current study, indicating that HIF-1$\alpha$ can regulate VEGF-A expression in glioma cells (Fig. 6J-K). ELISA analysis demonstrated that knockdown of HIF-1$\alpha$ suppressed concentration of VEGF-A in the medium conditioned from U87 and U251 cells (Fig. 5F). To be brief, these results demonstrate that ELK3 regulates VEGF-A expression and induces tubule formation by interacting with HIF-1$\alpha$.

### ELK3 inhibits tumor progression and angiogenesis in vivo

**Fig. 7:**
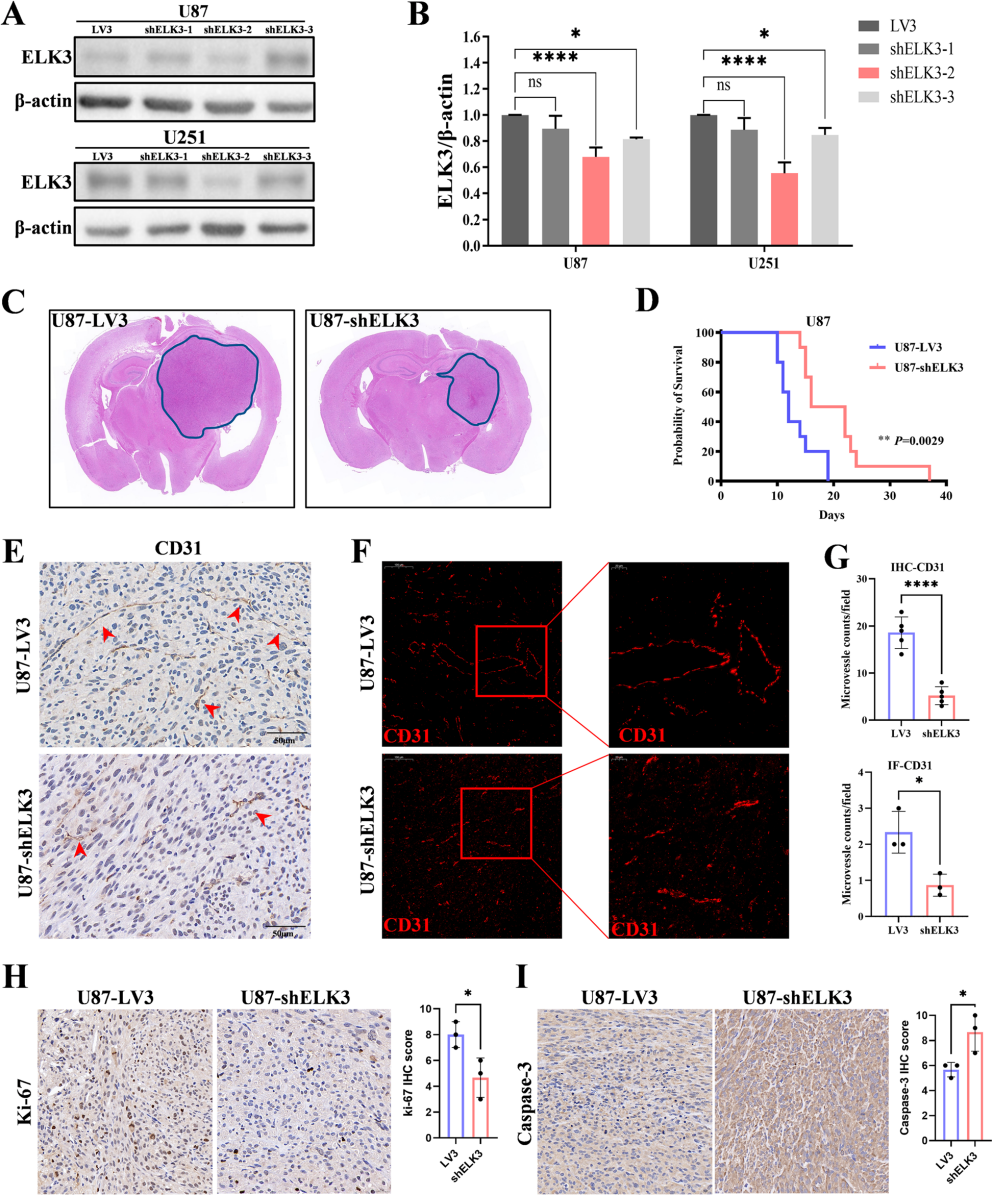
ELK3 inhibits tumor progression and angiogenesis in vivo. **A**,**B** Western blotting was used to confirm a significant reduction ELK3 expression after being infected with LV3 (sh control) or shELK3 lentiviruses in U87 and U251 cells. **C** Representative images of Glioma sections stained with H &E from mice whose tumors were formed by LV3-transduced U87 cells and shELK3-transduced U87 cells. **D** Kaplan-Meier survival curves of mice (n = 10/group) bearing U87 tumors formed by LV3-transduced U87 cells and shELK3-transduced U87 cells. * P <0.05. **E** CD31 immunohistochemical staining (arrow) indicates that silencing ELK3 inhibited angiogenesis in mice bearing U87 tumors. **F** CD31 immunofluorescence staining (red) shows that silencing ELK3 inhibited angiogenesis in mice bearing U87 tumors. **G** Quantification of CD31$+$ microvessel density in immunofluorescence and immunohistochemical staining. **H** Immunohistochemistry for ki-67 in the two group. **I** Immunohistochemistry for caspase-3 in the two group. All *p* values resulted from 2-sided statistical test, ns: not significant, and all experiments were repeated at least three times (*P<0.05, **P<0.01, ***P<0.001, ****P<0.0001)

To evaluate the effect of ELK3 in vivo, U87-shELK3 and U87-LV3(empty vector) cells were used to examine the effect of ELK3 on glioma tumor growth in vivo. First, lentivirus shELK3-2 was detected to have inhibited the expression of ELK3 (Fig. 7A-B). Then, U87-shELK3 and U87-LV3 GBM cells were intracranially injected into 6-week-old nude mice. The mice began to die 19 days after glioma transplantation. Their brain was isolated just before death for histopathological analysis. Histopathological and immunological analyses of the isolated tumors were performed to verify whether ELK3 knockdown affected in vivo tumor progression in nude mice. Results revealed significantly smaller tumor range in the U87-shELK3 glioma-bearing mice than in the U87-LV3 mice (Fig. 7C), and that the median survival of the U87-shELK3 glioma-bearing mice group was 40$\%$ longer than that in the U87-LV3 group (Fig. 7D). Moreover, CD31 immunohistochemistry and immunofluorescence of fixed tumor tissue sections showed that CD31 positive rate of the U87-shELK3 group was significantly lower than that of the U87-LV3 group (Fig. 7E-G). In addition, ki-67 and caspase-3 staining of fixed tumor tissue sections showed that ki-67 positive rate of the U87-shELK3 group was significantly lower than that of the U87-LV3 group whereas caspase-3 positive rate increased significantly (Fig. 7H-J). These results suggest that ELK3 knockdown can inhibit tumor progression and angiogenesis in vivo.

## Discussion

**Fig. 8:**
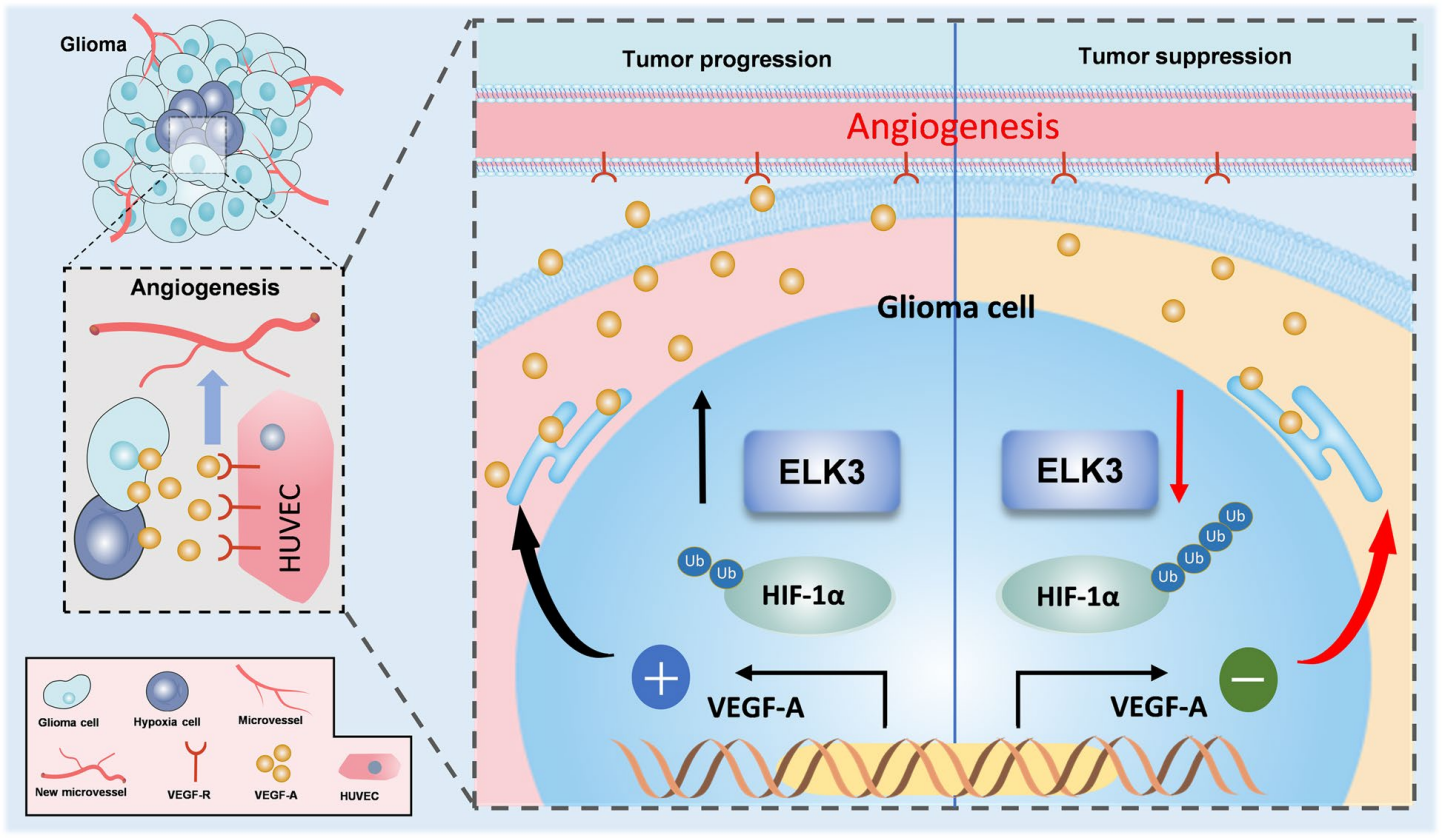
ELK3 promotes glioma angiogenesis progression by activating the HIF-1$\alpha$/VEGF-A signaling axis. In normal glioma, the growth rate of blood vessels inside the tumor is slower than that of tumor cells, and the cells show transient hypoxia. ELK3 is highly expressed in tumor cells, which promotes the expression and release of cytokine VEGF-A by regulating HIF-1$\alpha$ expression, and promotes new microangiogenesis outside the cell and promotes tumor progression. When knockdown ELK3, a decrease in ELK3’s ability to bind to HIF-1$\alpha$ results in an increase in HIF-1$\alpha$ ubiquitination, which suppresses VEGF-A transcript expression and release, inhibiting glioma angiogenesis and slowing tumor progression

Current studies revealed that ELK3 can promote the occurrence and development of glioma and play a promoting role in glioma angiogenesis. In short, ELK3 regulates VEGF-A expression and secretion by ubiquitinating HIF-1$\alpha$, thereby controlling glioma angiogenesis. Firstly, ELK3 was found to be significantly upregulated in glioma specimens and the high expression level of ELK3 was correlated with poor prognosis. Various functional experiments were used to evaluate the role of ELK3 in the proliferation, apoptosis, migration, and invasion of glioma cells. ELK3 was shown to positively promote malignant phenotypes. A series of deletion, overexpression, and salvage function experiments were conducted to evaluate the production and angiogenesis of extracellular VEGF-A, in order to confirm that ELK3 promotes glioma angiogenesis by regulating the expression and secretion of VEGF-A. Meanwhile, ELK3 can regulate HIF-1$\alpha$ ubiquitination. It was ultimately discovered that ELK3 promotes glioma angiogenesis is progressing by activating the HIF-1$\alpha$/ VEGF-A signaling axis (Fig. 8).

The main characteristics of glioma are invasive growth and strong angiogenic ability, resulting in a high invasion and recurrence rate. This study found a significant negative correlation between the expression level of ELK3 and the postoperative survival time of patients, indicating that patients with higher levels of ELK3 expression in tumor tissue have poorer prognosis. This is consistent with a study by Liu et al. on the relationship between ELK3 expression and prognosis in glioma patients [22]. In this study, the authors confirmed that ELK3 is abnormally overexpressed in glioma, and it was confirmed that overexpression of ELK3 can promote the malignant transformation of glioma. However, the study only conducted bioinformatics analysis and simple cell phenotype validation, and did not involve the exploration of the correlation between ELK3 and the metabolic process of glioma. The current study, with functional experiments in vitro and in vivo, esp. demonstrated that ELK3 promoted tumor migration and invasion in glioma. This is similar to its role in other tumors, in squamous cell and pancreatic carcinoma, ELK3 gene knockdown can considerably hinder tumor growth [15, 23]. Studies on the role of ELK3 in tumor metastasis reported that ELK3 facilitates metastasis of pancreatic carcinoma, bladder cancer and breast cancer [23–25]. Nonetheless, the mechanism that underlies ELK3-promoted glioma progression is yet to be revealed.

Studies have shown that intertumoral neovascularization is an important feature of GBM [26]. With multiple angiogenic growth factors expressed in tumors, GBM has become one of the highly vascularized tumors [27]. Therefore, the mechanism underlying glioma angiogenesis should be elucidated to develop more effective therapeutic approaches to the treatment of gliomas with angiogenesis. ELK3, as a member of ternary complex factors (TCF), a subfamily of ETS domain transcription factors, can form ternary complexes with serum response factors (SRF) to regulate gene expression [28]. It is associated with the occurrence and progression of many types’ cancers. In recent years, most angiogenesis studies on glioma have focused on angiogenic factors and cytokines. However, in two phase-III studies, Bevacizumab, a VEGF-A blocker, was added to standard treatment (radiotherapy + Temozolomide) for newly diagnosed glioblastoma patients [29, 30]. In these studies, progression-free survival increased by 4 months without affecting overall survival significantly [31]. Additionally, Bevacizumab was associated with increased adverse events, entailing the necessity for new pharmacological approaches [4]. In the current study, we examined the role of ELK3 in promoting glioma angiogenesis and malignancy. ELK3 was upregulated in glioma tissues. Silencing ELK3 decreased glioma angiogenesis in vitro and in vivo by regulating the expression and secretion of VEGF-A. This has never been reported in previous studies.

Angiogenesis, vital to tumor growth and progression of gliomas, is involved in many processes, including, angiogenesis, vasculogenesis, and tumor stem cell transdifferentiation of glioma stem cells [32, 33]. Antiangiogenic therapies can normalize tumor vasculature and therefore improve treatment efficacy when co-administered with other therapies in other tumors [34]. Preclinical studies have shown that the first-line use of Bevacizumab, an anti-angiogenesis, fails to improve the overall survival in patients with newly diagnosed glioblastoma [4]. Therefore, it is particularly important and imperative to find new approaches to anti-angiogenic therapies. The current study has shown that silencing ELK3 can inhibit angiogenesis both in vitro and in vivo.

VEGF-A is one of the most important activators of angiogenesis. Many studies have shown that it is highly up-regulated in cancer cells. A decrease in VEGF-A in myeloid cells attenuated glioma progression and prolonged survival in an experimental glioma model of transgenic mice [35]. HOTAIR regulated VEGF-A expression in glioma cells with extracellular vesicles and then entered the endothelial cells [36]. In the current study, we have evidenced that VEGF-A is a downstream molecule critical for ELK3 and that ELK3 transcriptionally regulates VEGF-A expression and thereby indirectly promotes tube formation in vitro of HUVEC. What’s more, we have also explored the specific mechanism underlying the ELK3 regulation of VEGF-A.

Hypoxia, as one of the most effective triggers in a wide range of angiogenic stimulations, plays its role mainly by activating HIF-1 (hypoxia-inducible factor-1) and regulating VEGF-A expression [37]. Studies have shown that l deletion of VEGF-A in CD8+ T cells accelerated tumorigenesis while altering vascularization, suggesting that HIF-1$\alpha$/VEGF-A axis is an essential aspect of tumor immunity [38–40]. Based upon these findings, we may infer that HIF-1$\alpha$, as an upstream transcriptional factor of VEGF-A, has a manifest function in gliomas. We thus hypothesize that ELK3 can regulate VEGF-A expression by interacting with HIF-1$\alpha$. Our research shows that ELK3 transcriptionally regulated expression and secretion of VEGF-A via interacting with HIF-1$\alpha$. Therefore, we suggest that ELK3 can promote angiogenesis by regulating VEGF-A in gliomas.

Our findings suggest that ELK3 can promote angiogenesis in gliomas. However, this study also has certain limitations, as a clinical therapeutic drug targeting VEGF-A, bevacizumab has not been able to provide a certain experimental basis for future combination therapy with ELK3 in this study. In addition, the function of angiogenesis in the development of malignant tumors deserves to be investigated in tumor biology more comprehensively. Finally, drugs targeting ELK3 intervention need to be further constructed and combined with anti-vascular therapies to promote clinical application.

In conclusion, this study found that ELK3 was upregulated in glioma tissues and was hence associated with poor prognosis. More importantly, our findings indicate that ELK3 promotes the malignant progression of glioma by promoting VEGF-A expression and inducing angiogenesis via interacting with HIF-1$\alpha$. This study also describes a new mechanism of the ELK3/HIF-1$\alpha$/VEGF-A axis in glioma progression. Therefore, these findings suggest that ELK3 is a prognostic marker with a great potential for glioma angiogenesis and targeting ELK3 as a therapeutic strategy may improve the efficacy of anti-angiogenesis treatment.

## Conclusions

This study found that ELK3 was upregulated in glioma tissues and was hence associated with poor prognosis. More importantly, our findings indicate that ELK3 promotes the malignant progression of glioma by promoting VEGF-A expression and inducing angiogenesis via interacting with HIF-1$\alpha$. This study also describes a new mechanism of the ELK3/HIF-1$\alpha$/VEGF-A axis in glioma progression. Therefore, these findings suggest that ELK3 is a prognostic marker with a great potential for glioma angiogenesis and targeting ELK3 as a therapeutic strategy may improve the efficacy of anti-angiogenesis treatment.

## Data Availability

The data presented in the current study are available from the corresponding author on reasonable request.

## Abbreviations

ELK3: ETS Transcription Factor ELK3
HIF-1$\alpha$: Hypoxia Inducible Factor 1 Subunit Alpha
VEGF-A: Vascular Endothelial Growth Factor A
GFAP: Glial Fibrillary Acidic Protein
CD31: Platelet endothelial cell adhesion molecule-1
SOX2: SRY-Box Transcription Factor 2
HUVEC: Human Umbilical Vein Endothelial Cells
TMA: tissue microarrays
